# The Seraph 100^®^ Microbind Affinity Blood Filter Does Not Alter Levels of Circulating or Mucosal Antibodies in Critical COVID-19 Patients

**DOI:** 10.3390/antib13030065

**Published:** 2024-08-06

**Authors:** Tonia L. Conner, Pooja Vir, Eric D. Laing, Ian J. Stewart, Edward Mitre, Kathleen P. Pratt

**Affiliations:** 1 Department of Microbiology, Uniformed Services University of the Health Sciences, Bethesda, MD 20814, USA; tonia.conner@usuhs.edu (T.L.C.); eric.laing@usuhs.edu (E.D.L.); 2 The Henry Jackson Foundation for the Advancement of Military Medicine, Bethesda, MD 20814, USA; pooja.vir.ctr@usuhs.edu; 3 Department of Medicine, Uniformed Services University of the Health Sciences, Bethesda, MD 20814, USA; ian.stewart@usuhs.edu

**Keywords:** COVID-19, SARS-CoV-2, antibodies, mucosal, Seraph 100

## Abstract

PURIFY-OBS-1 is an observational study evaluating the safety and efficacy of Seraph 100^®^ Microbind Affinity Blood Filter (Seraph 100) use for COVID-19 patients with respiratory failure admitted to the intensive care unit (ICU). The Seraph 100 is a hemoperfusion device containing heparin-coated beads that can bind to, and reduce levels of, some circulating pathogens and inflammatory molecules. This study evaluated whether treatment with the Seraph 100 affected circulating and mucosal antibody levels in critically ill COVID-19 subjects. SARS-CoV-2 anti-spike and anti-nucleocapsid IgG and IgA levels in serum were evaluated at enrollment and on days 1, 4, 7, and 28 after Seraph 100 application, while anti-spike and nucleocapsid IgG, IgA, and secretory IgA levels in tracheal aspirates were evaluated at enrollment and on days 1, 2, 3, 7, and 28. Serum samples were also collected from the pre- and post-filter lines at 1 and 4 h following Seraph 100 application to evaluate the direct impact of the filter on circulating antibody levels. Treatment with the Seraph 100 did not alter the levels of circulating or mucosal antibodies in critically ill COVID-19 subjects admitted to the ICU.

## 1. Introduction

The COVID-19 pandemic resulted in 103.4 million cases and 1.2 million deaths in the United States [1], yet it also paved the way for scientific innovation for the treatment and protection against viral infections [2]. Between 2020 and 2021, the mortality rate of COVID-19 patients admitted to the intensive care unit (ICU) was almost 50% [3,4], leading to the expanded use of the emergency use authorization (EUA) by the Federal Drug Administration [5] to provide potentially lifesaving medical interventions [6]. One of those interventions was the Seraph 100^®^ (Seraph 100) Microbind Affinity Blood Filter, a hemoperfusion device, approved for use in COVID-19 patients admitted to the ICU with respiratory failure [7]. Through the use of heparin-coated beads that solicit binding of pathogens, the Seraph 100 has been shown to reduce levels of some pathogenic bacteria and viruses in the blood, thereby showing promise to improve clinical outcomes. The potential benefits of the Seraph 100 in COVID-19 were hypothesized to be due to the possibility that it could reduce the SARS-CoV-2 viral load in the blood. Additional possibilities were that it might lower the risk of multiorgan failure and/or mitigate risks of coagulopathy by binding circulating inflammatory cytokines/chemokines and/or essential clotting factors as well [8,9,10].

Given the central role antibodies have in protection against severe disease from SARS-CoV-2 [11], in this study, we sought to characterize the impact that the Seraph 100 filter has on circulating SARS-CoV-2-specific antibody responses. Despite its emergence as a possible therapeutic adjunct for several inflammatory diseases [10,12,13,14,15,16], to date, only two studies have evaluated antibody levels during the course of Seraph 100 hemoperfusion [17,18]. In this study, we evaluated SARS-CoV-2 specific binding antibodies from serum and tracheal aspirate (TA) samples obtained before, during, and after initial treatment with the Seraph 100. We measured anti-SARS-CoV-2 spike and nucleocapsid (N) IgG and IgA levels from serum samples and TA samples. Additionally, in TA samples, we measured SARS-CoV-2 anti-spike and anti-N secretory IgA (SIgA), which is found only in mucosal secretions and is indicative of a local mucosal immune response [19].

Samples were obtained from severe COVID-19 subjects enrolled in the PURIFY-OBS-1 study, a prospective observational study to evaluate clinical outcomes following hemoperfusion with the Seraph 100. Additional goals were to analyze multiple analytes in serial blood, urine, and saliva or endotracheal aspirates from samples acquired over up to 28 days after ICU admission.

## 2. Materials and Methods

### 2.1. Human Subjects

Study participants were enrolled in PURIFY-OBS-1, a prospective observational study evaluating the safety and efficacy of the Seraph 100^®^ Microbind Affinity Blood Filter (Seraph 100, ExThera Corp., Martinez, CA, USA) in hospitalized patients with severe COVID-19 [13]. The Seraph 100 was utilized under an emergency use authorization from the FDA. Study criteria and institutional review board [20] approvals were detailed previously by Chitty et al. [13]. Briefly, all participants provided informed consent and the protocol was approved by the Advarra Institutional Review Board under protocol # Pro00047577.

Serum and TA samples were collected upon enrollment (baseline = study day 0). The first Seraph 100 application (typically ~4 h duration) was at the discretion of the attending physician, a median of 3.6 days following enrollment (Supplemental Table S1); this was referred to as study day 1. To evaluate the direct impact of the filter, blood samples were also drawn simultaneously from the pre- and post-filter lines at hours 1 and 4 following filter application. Additional blood samples were collected on study days 4, 7, and 28 as feasible. Tracheal aspirates were collected at baseline, and on study days 1, 2, 3, 4, 7, and 28 when possible. Samples were frozen in aliquots to avoid multiple freeze–thaw cycles and thereby enable accurate testing for multiple analytes.

### 2.2. Quantitative Binding Antibody Assays

Serum and TA aliquots were stored at −80 °C and then thawed at 4 °C overnight prior to testing. Serum samples were divided into further aliquots after thawing and then heat-inactivated for 30 min at 60 °C. These samples were diluted in phosphate-buffered saline (PBS) at 1:400, 1:4000, and 1:40,000 for IgG testing and at 1:400 and 1:4000 for IgA testing. TA samples were vortexed for 1 min after thawing and then centrifuged at 16,000× *g* for 10 min at 4 °C. Samples with high viscosity were centrifuged at 16,000× *g* for an additional 10 min to separate cellular material from the supernatant. TA supernatants were transferred to a new tube and heat-inactivated for 30 min at 60 °C. TA samples were diluted in PBS at 1:80 and 1:160 for IgG, 1:20 and 1:40 for IgA, and 1:5 and 1:10 for SIgA [21].

Binding antibodies were measured against the wild-type Wuhan-1 SARS-CoV-2 spike and nucleocapsid (N) proteins using a microsphere-based multiplex immunoassay (MMIA). The S glycoprotein was expressed as a prefusion stabilized ectodomain trimer and sourced from Curia (Albany, NY, USA), while the N protein was sourced from RayBioTech (Peachtree Corners, GA, USA). Each protein (15 μL S and 15 μL N) was coupled in separate reactions to 100 μL of magnetic carboxylated beads (Luminex, Austin, TX, USA) as previously described [22,23]. A 1:100 master mix of coupled beads/PBS was added to each well of a 96-well plate followed by 100 μL of diluted TA or serum sample. TA samples were rocked at room temperature for 45 min at 700 rpm, while serum samples were rocked at 900 rpm for 45 min. The plates were then washed with PBS plus 0.05% Tween 20 three times. 

Biotinylated cross-adsorbed goat anti-human IgG (Invitrogen, Waltham, MA, USA) and goat anti-human IgA (Invitrogen) were used as detection antibodies diluted at 1:5000 in PBS for both serum and TA assays. Goat anti-human SIgA (MyBioSource, San Diego, CA, USA) was biotinylated using a biotinylation kit (Abcam, Cambridge, UK), and also diluted at 1:5000 in PBS and used for detection of SIgA in TA samples. Each detection antibody was incubated with the samples at room temperature for 45 min, with TA samples rocking at 700 rpm and serum samples rocking at 900 rpm. After this incubation and three washes with PBS plus 0.05% Tween 20, a 1:1000 dilution of streptavidin-phycoerythrin (Invitrogen) was added to each well for another 45 min. After three more washes with PBS plus 0.05% Tween 20, 100 μL of with PBS plus 0.05% Tween 20 was added to each well, and incubated for 10 min while rocking at 700 rpm for TA samples and serum 900 rpm. At the end of incubation, median fluorescence intensity (MFI) was measured using a Bio-Plex 200 HTF multiplex system (BioRad, Hercules, CA, USA). For serum samples, anti-spike and anti-N IgG levels were interpolated using an internal reference standard calibrated to the Human SARS-CoV-2 Serology Standard for binding antibodies (Frederick National Laboratory for Cancer Research, Frederick, MD, USA). In the absence of calibrated standards for anti-spike and anti-N IgA binding antibody units, IgA from serum samples is reported in arbitrary binding units (AU/mL). TA samples were interpolated using a standard curve generated using known concentrations of purified human IgG, IgA, and SIgA coupled to magnetic microspheres and reported in arbitrary binding units (AU/mL).

### 2.3. Statistical Analysis

Comparisons between three or more groups were conducted using the nonparametric Kruskal–Wallis test followed by Dunn’s test for multiple comparisons. Friedman’s test with Dunn’s test for multiple comparisons was used for repeated measurements of three or more groups. Descriptive statistics were determined using Graph Pad Prism Software version 10 and provided geometric means (GM), medians, and interquartile ranges (IQR).

## 3. Results

### 3.1. Participant Demographics

Thirty-three subjects were enrolled in the study upon admission to the ICU for severe COVID-19. Of the entire cohort, 15 participants survived (45.5%) and 18 did not survive (54.5%). The majority of the participants were male (63.6%) and the median age was 45.0 (range 20–68; IQR 32.0–54.0, Table 1). The mean body mass index was slighter higher for the nonsurvivors at 36.3 (range 24.0–70.9; IQR 31.0, 43.3) compared to the survivors at 32.0 (range 23.8–51.2; IQR 28.9, 43.9), but these differences were not statistically significant. Detailed clinical data from the cohort are provided in Supplemental Table S1.

### 3.2. Serum and Mucosal Antibody Levels in the Days after ICU Admission

All subjects produced detectable serum and mucosal IgG and IgA antibodies against SARS-CoV-2 spike and N proteins. Geometric mean levels of serum anti-spike IgG were 746.7 BAU/mL and anti-spike IgA were 252.5 AU/mL (Supplemental Figure S1). While levels of serum antibodies varied during the two weeks after ICU admission, these levels appeared to stabilize around the geometric mean for each subject between three and seven weeks after diagnosis. While mucosal antibody levels also varied widely in the first two weeks, the majority of samples tested between three and seven weeks after diagnosis were below the geometric means (Supplemental Figure S2). Of note, levels of mucosal anti-spike and anti-N IgG, IgA, and SIgA antibodies were all markedly higher than levels our group measured in a separate study of saliva samples from individuals with mild to moderate outpatient COVID-19 disease [24].

### 3.3. Serum Antibody Levels Are Not Affected by Treatment with the Seraph 100 

**Figure 1:**
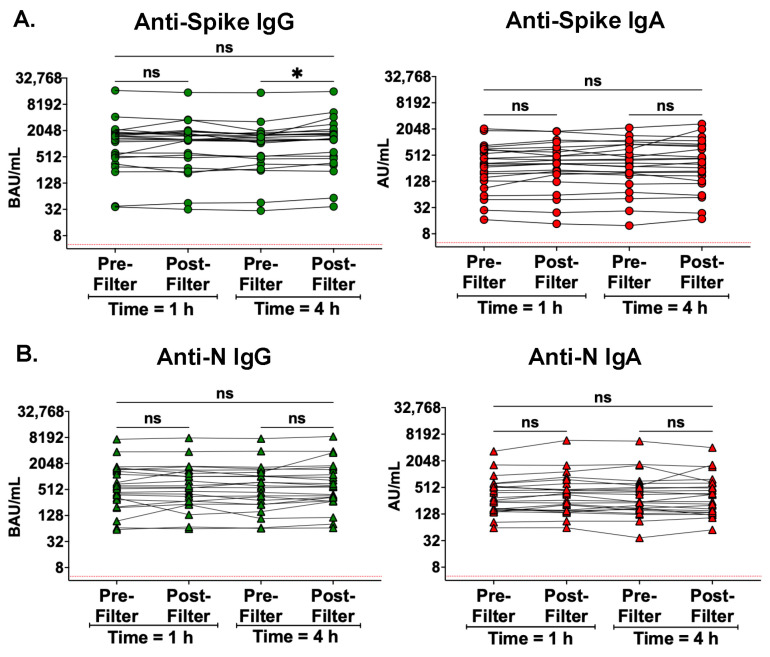
There were no differences between pre- and post-filter serum antibody levels during treatment with the Seraph 100. Antibody levels were measured both entering and then exiting the Seraph 100 filter at two time points: time = 1 hour (h) and time = 4 h following filter application. Serum antibody levels from participants with samples from all four timepoints, i.e., at 1 h pre/post filter and at 4 h pre/post-filter (n = 23), were analyzed using Friedman’s test with Dunn’s test for multiple comparisons. (**A**) Anti-spike IgG and IgA levels, and (**B**) anti-N IgG and IgA levels. The red dotted line represents the lower limit of quantitation for this assay; ns, not significant; * *p* < 0.05.

Anti-spike and anti-N IgG and IgA levels were measured in serum collected immediately before (pre-filter) and after (post-filter) entering the Seraph 100 at both 1 and 4 h after initial filter application to evaluate the immediate impact of the filter on serum antibodies. At both timepoints, no reductions in antibody levels were observed across the filter (Figure 1). A modest significant increase (1.2-fold, *p* = 0.0129) was observed in anti-spike IgG in the post-filter sample at hour 4 but not at hour 1 (Figure 1). 

**Figure 2:**
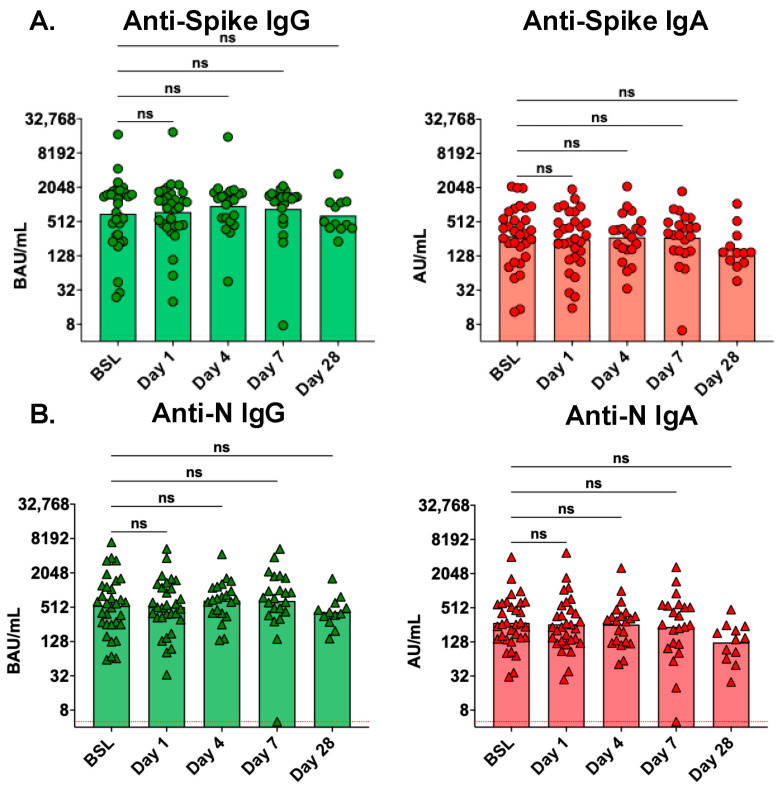
Serum antibody levels were not affected by treatment with the Seraph 100 Filter. Serum antibody levels of (**A**) anti-spike IgG and IgA and (**B**) anti-N IgG and IgA at baseline (BSL, n = 32), day 1 (n = 30), day 4 (n = 21), day 7 (n = 22), and day 28 (n = 12). The red dotted line represents the lower limit of quantitation for this assay. Serum antibody levels at later time points were compared to baseline levels using Kruskal–Wallis analysis with Dunn’s multiple comparison test; ns, not significant.

We next evaluated whether Seraph 100 hemoperfusion was associated with reduced levels of circulating antibodies in the days following initial treatment. There were no differences in the levels of serum anti-spike or anti-N IgG and IgA antibodies at 1, 4, or 7 days following Seraph 100 treatment compared to baseline (pre-Seraph treatment) levels (Figure 2). A modest, nonstatistically significant decrease was observed in all antibody levels at day 28 following Seraph 100 treatment (Figure 2). To enable statistical analysis by repeated measures, we next evaluated longitudinal serum antibody levels only among the nine participants who had serum collected at all five timepoints (Supplemental Figure S3). Comparisons were made between each timepoint as well as between baseline and day 28. Again, no differences were observed in the levels of any serum antibodies between baseline and days 1, 4, or 7. Statistically significant decreases were observed in serum levels of anti-spike IgA (day 7 vs. day 28, 1.5-fold, *p* = 0.0365, and baseline vs. day 28, 2.8-fold, *p* = 0.0231), anti-N IgG (baseline vs. day 28, 2.7-fold, *p* = 0.0143), and anti-N IgA (baseline vs. day 28, 2.8-fold, *p* = 0.0143) between baseline and day 28, while anti-spike IgG decreased 2.0-fold, but this did not reach statistical significance (*p* = 0.0854). 

### 3.4. Mucosal Antibody Levels Are Not Affected by Treatment with the Seraph 100

**Figure 3:**
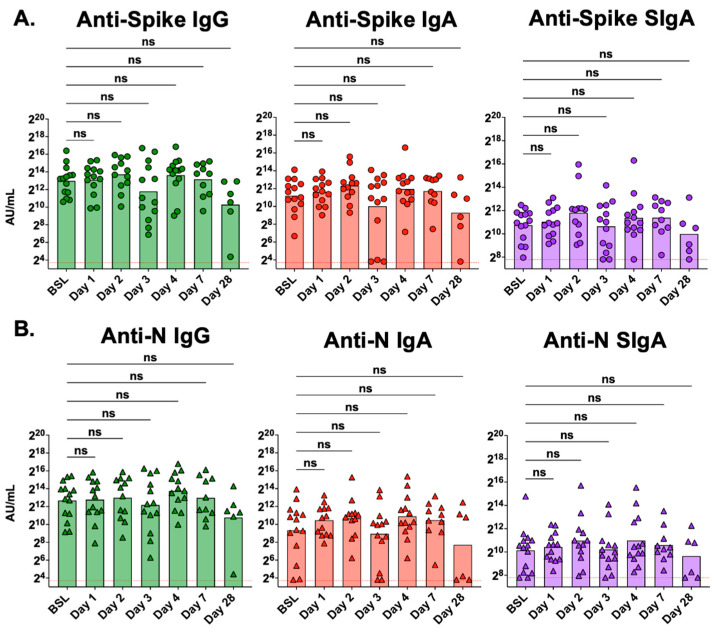
Mucosal antibody levels were not affected by treatment with the Seraph 100 Filter. (**A**) Mucosal antibody levels of anti-spike and (**B**) mucosal antibody levels of anti-N IgG, IgA, and SIgA at baseline (BSL, n = 14), day 1 (n = 13), day 2 (n = 12), day 3 (n = 13), day 4 (n = 13), day 7 (n = 10), and day 28 (n = 6) from samples collected during the study. The red dotted line represents the lower limit of quantitation for this assay. Mucosal antibody levels were compared to baseline levels using Kruskal–Wallis analysis with Dunn’s multiple comparison test; ns, not significant.

In Figure 3, we compare antibody levels in tracheal aspirate samples collected at baseline to those collected on study days 1, 2, 3, 4, 7, and 28. No statistically significant changes were observed in mucosal antibody levels of either anti-spike or anti-N IgG, IgA, or SIgA at any timepoint compared to baseline (pre-Seraph 100 treatment). Statistical analysis was also conducted using repeated measures for the five participants with samples collected at baseline and days 1, 3, and 7 after Seraph 100 treatment. This analysis also demonstrated no significant changes in mucosal antibody levels in the days following Seraph 100 treatment (Supplemental Figure S4).

### 3.5. No Differences between Peak Serum or Peak Mucosal Antibody Levels between Nonsurvivors and Survivors

**Figure 4:**
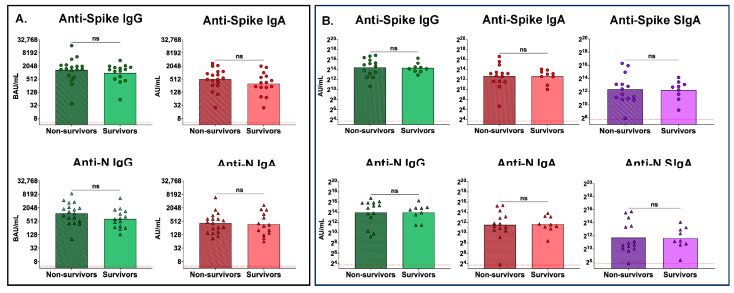
There were no differences in peak serum or mucosal antibody levels between nonsurvivors and survivors. (**A**) Peak serum antibody levels of anti-spike and anti-N IgG and IgA in nonsurvivors (n = 15) and survivors (n = 18) and (**B**) peak mucosal levels of anti-spike and anti-N IgG, IgA, and SIgA in nonsurvivors (n = 14) and survivors (n = 9). Red dotted line represents the lower limit of the assay. Comparisons were made using the Mann–Whitney test; ns, not significant.

To assess if there were differences between antibody levels of nonsurvivors and survivors, we compared the peak serum antibody levels between these groups (Figure 4). No differences were observed between nonsurvivors and survivors with regards to peak serum anti-spike IgG or IgA, or peak serum anti-N IgG or IgA, antibody levels (Figure 4A). Similarly, no significant differences were observed between survivors and nonsurvivors with respect to peak mucosal anti-spike or anti-N IgG, or peak mucosal IgA or SIgA, antibody levels (Figure 4B).

### 3.6. No Differences between Peak Serum or Peak Mucosal Antibody Levels between Males and Females

**Figure 5:**
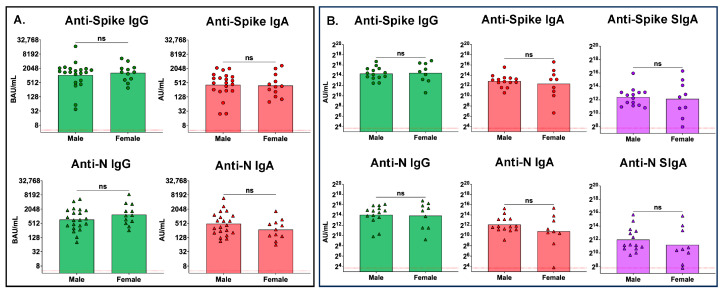
There were no differences in peak serum or mucosal antibody levels between males and females. (**A**) Peak serum antibody levels of anti-spike and anti-N IgG and IgA in males (n = 21) and females (n = 9) and (**B**) peak mucosal levels of anti-spike and anti-N IgG, IgA, and SIgA in nonsurvivors (n = 14) and survivors (n = 9). The red dotted line represents the lower limit of quantitation for this assay. Comparisons between male and female participants were made using the Mann–Whitney test; ns, not significant.

We compared the peak serum (Figure 5A) and mucosal (Figure 5B) antibody levels between males and females in the cohort. No sex-associated differences in either serum or mucosal peak antibody levels were observed.

## 4. Discussion

The Seraph 100 has been shown to reduce levels of some bacteria and viruses in the bloodstream of patients quickly after use, indicating that it may help to stabilize patients with septic shock [10,12,18,25,26]. This evidence supported the approval of the EUA in 2020 that allowed the Seraph 100 filter to be used to treat severe COVID-19 patients admitted to the ICU [7]. Since then, several studies have examined the impact of the Seraph 100 on the removal of N protein in the blood, impact on survival and mortality, and removal of inflammatory markers from the blood [13,14,16,27,28].

To date, however, only two studies have specifically reported on antibody levels in patients treated with the Seraph 100 [17,18]. One directly measured the amount of anti-spike IgG in the blood from a single pediatric patient with severe COVID-19 [17], and the other measured total immunoglobulin levels before and after treatment among fifteen subjects [18]. Both studies found no clear impact on circulating antibody levels. While promising, the data provided in these studies were limited regarding potential effects of Seraph 100 hemoperfusion on pathogen-specific antibodies.

In this study, we analyzed samples from the PURIFY-OBS-1 study to evaluate not only the immediate impact of the Seraph 100 on serum and mucosal antibody levels, but also the kinetics of anti-spike and anti-N antibodies in severely infected COVID-19 patients admitted to the ICU over a 28-day period. Our data demonstrate that the Seraph 100 filter reduces neither circulating nor mucosal antibody levels. Serum IgA and IgG antibody levels against spike and N proteins measured in post-filter serum samples were not reduced compared to pre-filter samples. Further, serum antibody levels remained fairly constant over the first 7 days after Seraph 100 treatment. Of note, repeated-measures analyses demonstrated modest decreases in serum levels of anti-spike IgA, anti-N IgG, and anti-N IgA at day 28 after filter application compared to baseline (pre-Seraph 100) levels. We suspect that this was due to natural decreases over time from peak antibody levels, consistent with another study that found that viral-specific antibody levels decreased modestly by 30 days after ICU admission [29]. An alternative explanation would be that Seraph-100 treatment causes reductions in antibody levels that manifest weeks after Seraph-100 treatment. While we believe that this is unlikely, future studies with the Seraph-100 should include ICU patients not treated with the device to decisively determine whether the Seraph-100 can cause a delayed reduction in antibody levels.

In addition to analyzing serum samples to quantify changes in circulating antibody levels, we obtained repeated measures of anti-spike and anti-N IgG, IgA, and SIgA antibody levels in tracheal aspirates. We observed no differences in mucosal antibody levels related to the use of the Seraph 100 over the course of the study. This finding was observed both in the entire cohort as well as in those for whom we conducted repeated measures analyses for all timepoints. The finding of persistently elevated mucosal antibodies in critically ill patients with COVID-19 has been implicated as a possible cause for increased damage to the pulmonary tissues [30,31,32].

While not a primary goal of this study, we also assessed whether peak serum or mucosal antibody levels were associated with survival in this cohort. We did not observe statistically significant differences in peak serum or mucosal antibody levels between survivors and nonsurvivors. While several studies have shown increased mortality among patients with low anti-spike IgG responses at time of ICU admission, others have found no correlation between antibody levels and mortality, similar to our findings [33,34,35].

Of note, in the Prospective Assessment of SARS-CoV-2 Seroconversion (PASS) study, our group recently measured mucosal antibody levels in 15 unvaccinated individuals with mild to moderate outpatient COVID-19 disease one month after diagnosis using the same microsphere-based multiplex immunoassay as in this study [24]. In comparison to results obtained in the PASS study, mucosal antibody levels obtained in critically ill participants in this (PURIFY-OBS-1) study were markedly higher. Specifically, geometric means of mucosal anti-spike IgG, IgA, and SIgA were 59-fold, 24-fold, and 12.8-fold higher, respectively, in the PURIFY-OBS-1 cohort of individuals with severe COVID-19, and geometric means of mucosal anti-N IgG, IgA, and SIgA were 216-fold, 44-fold, and 13.3-fold higher. While not a direct comparison, as mucosal antibodies in the PASS study were measured in saliva samples obtained from passive drool, and mucosal antibodies in this study were measured on tracheal aspirates, these findings suggest that critically ill patients produce far greater amounts of mucosal antibodies than individuals with mild to moderate outpatient infections. These results are supported by other studies that have identified different antibody [35,36] and B cell profiles [37,38,39] in mild versus severe COVID-19, with greater inflammatory responses in the lungs of those with severe disease [38,39].

While females typically produce higher levels of antibodies, particularly IgG, in response to infection and vaccination [40,41,42], we did not detect any influence of sex on peak serological or mucosal antibodies. It is possible that in critical illness sex factors play less of a role in driving antibody responses. Alternatively, given the relatively small number of total individuals evaluated in this study (females n = 12 and males n = 21), it is possible that the study was simply underpowered to detect small differences in antibody levels based on sex.

In this study, both IgA and SIgA levels are reported for tracheal aspirate samples. IgA is a measurement of total antigen-specific IgA, since the detection antibody is against the heavy chain of IgA, whereas the antibody used to assess SIgA levels only detects the subset of IgA that is SIgA. We are unable to determine what percentage of total antigen-specific IgA is antigen-specific SIgA as our mucosal assays, to include mucosal IgG assays, do not determine absolute quantitation of antibody concentrations. It is also not possible to make comparisons between levels of IgA and SIgA using these assays.

This study does present some limitations. Due to the severity of illness in the PURIFY-OBS-1 cohort, multiple medical interventions were employed in attempts to increase survival. These interventions could have affected both serum and mucosal antibody responses. Another limitation of this study is the relatively small size of the cohort. As the study only had 33 participants, it was not powered to detect small but significant changes in antibody levels. Additionally, while several races/ethnicities were represented, most participants were white, and this could potentially affect the generalizability of the findings. We also were unable to test antibody levels in a control group of ICU-admitted subjects not treated with the Seraph 100 to use as a comparison to the Seraph 100-treated group. Rather, we utilized pre-Seraph filtration samples as controls for post-filtration samples. A final limitation is that the findings in this study may not be applicable to all SARS-CoV-2 variants. While SARS-CoV-2 variants were not identified in this study, most participants were enrolled when the Delta (B.1.617.2) variant was predominant. Thus, the findings of this study may not necessarily apply to non-Delta SARS-CoV-2 variants.

## 5. Conclusions

Critically ill participants in the PURIFY-OBS-1 study developed high serum and mucosal antibodies to SARS-CoV-2 spike and N proteins, with particularly higher mucosal IgG IgA and SIgA levels compared to levels in saliva from nonsevere COVID-19 subjects [24]. We showed that the Seraph 100 does not negatively impact serum or mucosal antibody levels in patients with severe COVID-19. Levels of mucosal antibodies remained elevated through 28 days post-Seraph 100 filter application, while circulating antibodies were high initially and then decreased modestly over the subsequent 28 days. Levels of serum and mucosal antibodies did not correlate with survival, and we did not observe differences in antibody levels between sexes. The results of this study demonstrate that the Seraph 100 filter does not directly remove or otherwise markedly affect circulating or mucosal antibodies against SARS-CoV-2.

## Figures and Tables

**Table 1:** Demographics of participants in study.

	All	Survivors	Non-Survivors
**Total study cohort**	33/33 (100%)	15/33 (45.5%)	18/33 (54.5%)
**Sex**			
Male	21/33 (63.6%)	11/15 (73.3%)	10/18 (55.6%)
Female	12/33 (36.4%)	4/15 (26.7%)	8/18 (44.4%)
**Race**			
Asian	1/33 (3.0%)	1/15 (6.7%)	0/18 (0.0%)
Black	2/33 (6.1%)	0/15 (0.0%)	2/18 (11.1%)
Latin American	1/33 (3.0%)	1/15 (6.7%)	0/18 (0.0%)
Other	4/33 (12.1%)	1/15 (6.7%)	3/18 (16.7%)
Unknown	3/33 (9.1%)	0/15 (0.0%)	3/18 (16.7%)
White	22/33 (66.7%)	12/15 (80.0%)	10/18 (55.6%)
**Ethnicity**			
Hispanic	14/33 (42.4%)	5/15 (33.3%)	9/18 (50.0%)
Non-Hispanic	13/33 (39.4%)	6/15 (40.0%)	7/18 (38.9%)
Unknown	6/33 (18.2%)	4/15 (26.7%)	2/18 (11.1%)
**Age, median (IQR)**	45.0 (32.0, 52.0)	45.0 (32.0, 58.0)	45.5 (30.5, 52.3)
**Body Mass Index, median (IQR)**	35.5 (29.4, 43.1)	32.0 (28.9, 43.9)	36.3 (31.6, 43.1)

## Data Availability

The raw data supporting the conclusions of this article will be made available by the authors on reasonable request.

## Supplementary Materials

The following supporting information can be downloaded at: https://www.mdpi.com/article/10.3390/antib13030065/s1, Table S1: Clinical data of participants in study and subset of survivors and nonsurvivors; Figure S1: Serum antibody levels measured in days after ICU admission; Figure S2: Mucosal antibody levels measured in days after ICU admission; Figure S3: Decreases were observed in serum anti-spike IgA, anti-N IgG, and anti-N IgA levels at day 28 post-ICU admission compared to baseline (ICU admission); Figure S4: No differences were observed in mucosal anti-spike or anti-N IgG, IgA, or SIgA through day 7 after ICU admission.
