# Supplementary material for: The Seraph 100® Microbind Affinity Blood Filter Does Not Alter Levels of Circulating or Mucosal Antibodies in Critical COVID-19 Patients
Source: Antibodies (Basel). 2024 Aug 6;13(3):65. doi: 10.3390/antib13030065 (PMC11348046; doi:10.3390/antib13030065)
Supplement: Supplementary file 1 [file antibodies-13-00065-s001.zip › antibodies-3062013-supplementary.pdf]

Supplemental Table S1: Clinical data of participants in study

	All	Survivors	Non-Survivors
		Median (IQR)	
Days since COVID-19 diagnosis upon admission to ICU	7.0 (2.5 ,12.0)	6.0 (3.0, 10.0)	9.0 (2.0 ,13.0)
Days from ICU admission until first Seraph treatment	3.6 (1.6, 5.7)	2.6 (1.5, 5.7)	4.4 (3.5, 7.4)
Days from ICU admission until death among subjects that died	N/A	N/A	36.2 (16.3, 84.4)
Days from ICU admission until discharge among subjects that survived	N/A	28.0 (9.0, 50.0)	N/A
Days the patient was alive and not in the ICU during the study	0.0 (0.0, 9.5)	12.0 (0.0, 20.0)	0.0 (0.0, 0.0)
Days the patient was alive and not on vasopressors during the study	15.0 (7.5, 28.0)	24.0 (24.0, 28.0)	8.0 (4.8, 12.8)
Days the patient was alive and not on mechanical ventilation during the study	6.0 (1.5, 22.5)	18.0 (6.0, 26.0)	3.5 (1.0, 8.0)
Apache II score	13.0 (8.5, 22.0)	13.0 (8.0, 22.0)	12.5 (8.8, 22.8)
Charlson Comorbidity Index	1 (0, 1)	1 (0, 1)	1 (0, 2)
		n%	
Treated with corticosteroids	33/33 (100%)	15/15 (100%)	18/18 (100%)
Treated with remdesivir	26/33 (78.8%)	12/15 (80.0%)	14/18 (77.8%)
Received mechanical ventilation at any point	30/33 (90.9%)	12/15 (80.0%)	18/18 (100%)
Received mechanical vent within first 24 hours of Seraph 100 treatment	25/33 (75.8%)	8/15 (53.3%)	17/18 (94.4%)
Received RRT at any point	18/33 (54.5%)	8/15 (53.3%)	10/18 (55.6%)
Received RRT within first 24 hours of Seraph 100 treatment	9/33 (27.3%)	4/15 (26.7%)	5/18 (27.8%)
Received ECMO at any point	16/33 (48.5%)	5/15 (33.3%)	11/18 (61.1%)
Received ECMO within first 24 hours of Seraph 100 treatment	15/33 (45.5%)	5/15 (33.3%)	10/18 (55.6%)

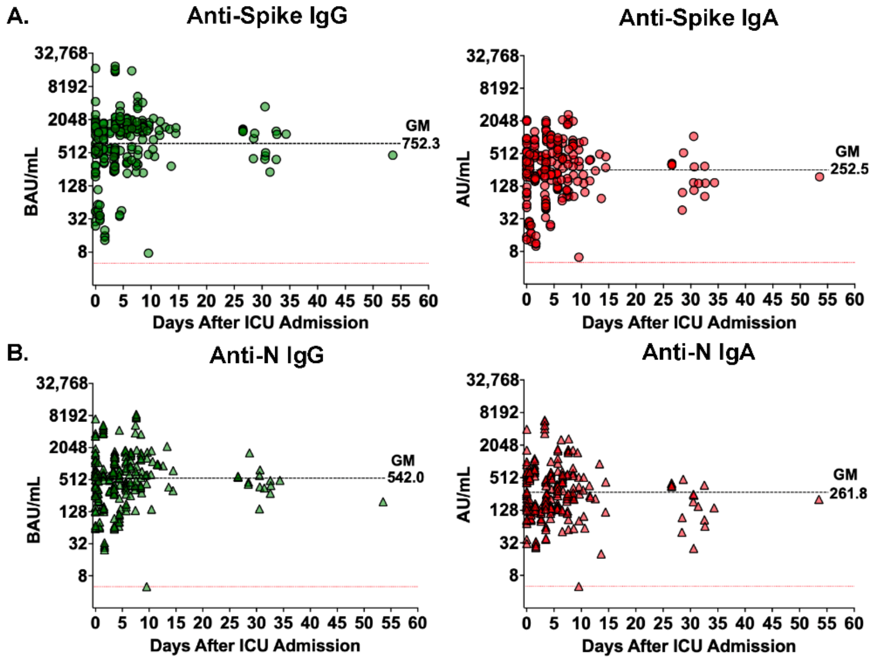

Supplemental Figure S1: Serum antibody levels measured in days after ICU admission. A) Serum antibody levels of anti-spike and B) serum antibody levels of anti-N IgG and IgA shown in days after ICU admission. The red dotted line represents the lower limit of quantitation for this assay.

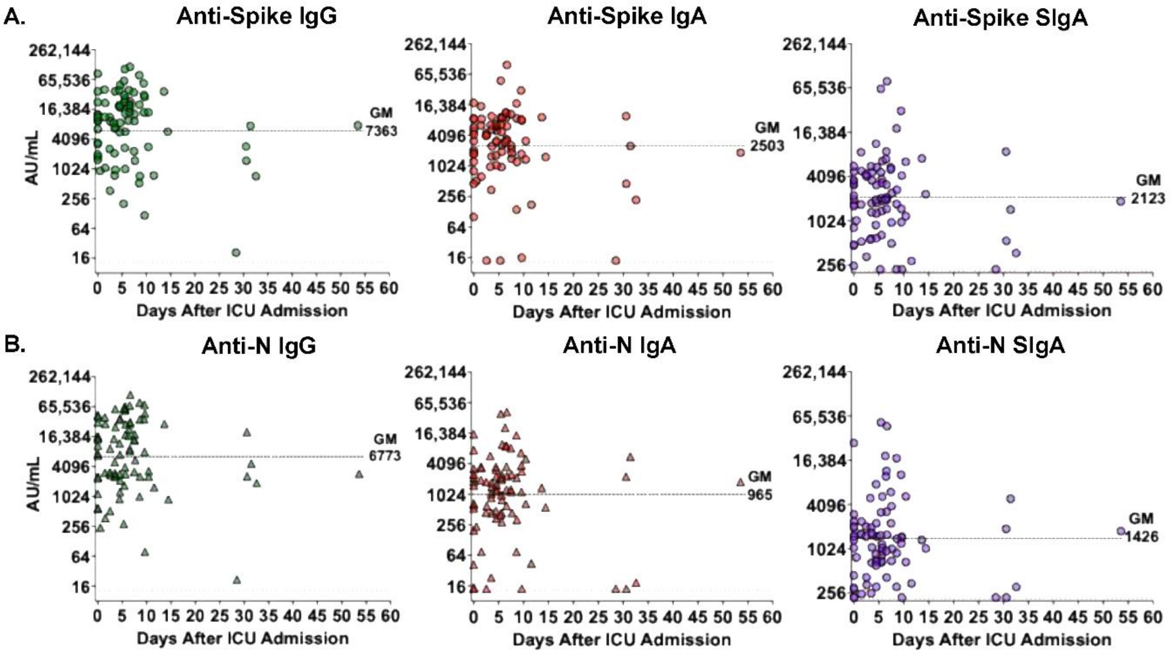

Supplemental Figure S2: Mucosal antibody levels measured in days after ICU admission. A) Mucosal antibody levels of anti-spike and B) mucosal antibody levels of anti-N IgG, IgA and SIgA measured in days after ICU admission. The red dotted line represents the lower limit of quantitation for this assay.

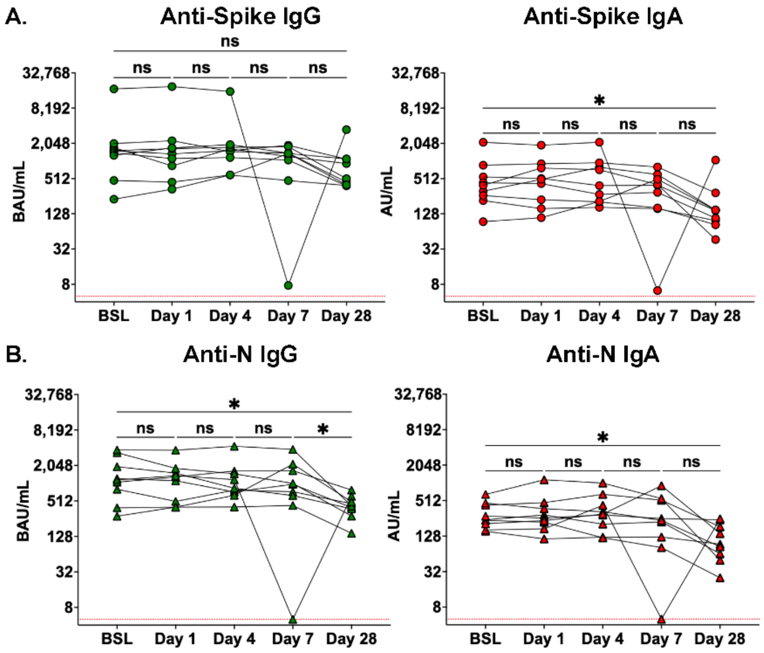

Supplemental Figure S3: Decreases were observed in serum anti-spike IgA, anti-N IgG and anti-N IgA levels at Day 28 post-ICU admission compared to baseline (ICU admission). A) Anti-spike and B) anti-N IgG and IgA levels in serial serum samples collected from 9 participants with samples collected over 28 days, analyzed using Friedman's test with Dunn's multiple comparison test. The red dotted line represents the lower limit of quantitation for this assay. ns, not significant; * $p < 0.05$.

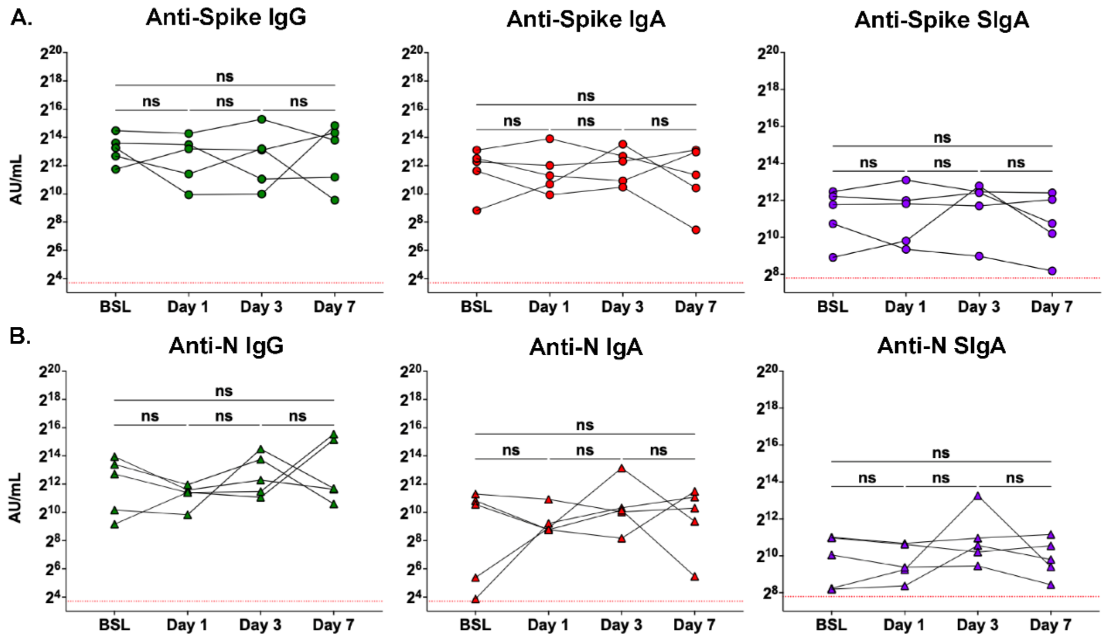

Supplemental Figure S4: No differences were observed in mucosal anti-spike or anti-N IgG, IgA or SIgA through Day 7 after ICU admission. A) Anti-spike and B) anti-N IgG, IgA, and SIgA in serial TA samples collected from 5 participants with samples collected over 7 days, analyzed using Friedman's test with Dunn's multiple comparison test. The red dotted line represents the lower limit of quantitation for this assay. ns, not significant.
